# Case study for the assessment of the biogeophysical effects of a potential afforestation in Europe

**DOI:** 10.1186/1750-0680-8-3

**Published:** 2013-02-01

**Authors:** Borbála Gálos, Stefan Hagemann, Andreas Hänsler, Georg Kindermann, Diana Rechid, Kevin Sieck, Claas Teichmann, Daniela Jacob

**Affiliations:** 1 Max Planck Institute for Meteorology, Hamburg, Germany; 2 Climate Service Center – eine Einrichtung am Helmholtz-Zentrum Geesthacht, Hamburg, Germany; 3 IIASA, International Institute for Applied Systems Analysis, Laxenburg, Austria

**Keywords:** Land cover change, Afforestation, Biogeophysical feedbacks, Climatic extremes, Regional climate modelling

## Abstract

**Background:**

A regional-scale sensitivity study has been carried out to investigate the climatic effects of forest cover change in Europe. Applying REMO (regional climate model of the Max Planck Institute for Meteorology), the projected temperature and precipitation tendencies have been analysed for summer, based on the results of the A2 IPCC-SRES emission scenario simulation. For the end of the 21st century it has been studied, whether the assumed forest cover increase could reduce the effects of the greenhouse gas concentration change.

**Results:**

Based on the simulation results, biogeophysical effects of the hypothetic potential afforestation may lead to cooler and moister conditions during summer in most parts of the temperate zone. The largest relative effects of forest cover increase can be expected in northern Germany, Poland and Ukraine, which is 15–20% of the climate change signal for temperature and more than 50% for precipitation. In northern Germany and France, potential afforestation may enhance the effects of emission change, resulting in more severe heavy precipitation events. The probability of dry days and warm temperature extremes would decrease.

**Conclusions:**

Large contiguous forest blocks can have distinctive biogeophysical effect on the climate on regional and local scale. In certain regions of the temperate zone, climate change signal due to greenhouse gas emission can be reduced by afforestation due to the dominant evaporative cooling effect during summer. Results of this case study with a hypothetical land cover change can contribute to the assessment of the role of forests in adapting to climate change. Thus they can build an important basis of the future forest policy.

## Background

Climate change and its impacts on different spatial and temporal scales and sectors have been addressed by several international research projects in the last decade [1-3]. All regional climate projections agree that at the end of the 21st century, a warming is expected in all seasons over Europe. The spatial patterns of the temperature changes in summer indicate the largest increase in the Mediterranean region, Southern France and over the Iberian Peninsula, while less warming is projected over Scandinavia [4,5]. Annual precipitation changes show a north–south gradient over Europe, with increase in the north (especially in winter) and decrease in the south (especially in the Mediterranean area in summer).

The considerable enhancement of inter-annual variability of the European summer climate as well as the changes of the hydrological cycle can lead to higher probability of extremes compared to present-day conditions [4,6-11]. The frequency of warm/wet and warm/dry events is projected to increase while the cold extremes show a significant decrease by 2100 [12]. The Mediterranean and the South-East European regions are the most prone to higher risks of heat waves and prolonged dry spells [8,13]. Whereas in Northern to North-Eastern Europe the number of days with intense precipitation is very likely to increase, which can result in a rise in flood frequencies [8,14-16]. The Central-Mediterranean and Central-Western Europe seem to be especially vulnerable to increases in both summer drought and flood [12,14].

Climate change affects the key sectors such as hydrological systems, infrastructure, human health, agriculture and forestry. Changes of the climatic means and extremes already show impacts on land cover that are expected to be more severe under future climate conditions. Drought periods and other extremes are responsible for a significant share of agricultural losses in Europe. Impacts of severe droughts on the composition, structure, and biogeography of forests have been detected worldwide in the recent decades [17,18]. On the lower limit of the forest distribution [19,20] ecological models expect growth decline and mass mortality of many zonal tree species whose distributions are limited primarily by recurrent droughts [21,22]. This phenomenon is not typical in humid areas of Europe [23].

Land cover in turn interacts with the atmosphere, thus it has an important role in climate regulation. Vegetation affects the physical characteristics of the land surface (biogeophysical feedbacks), which control the surface energy fluxes and hydrological cycle. Through biogeochemical processes, ecosystems alter the biogeochemical cycles and thereby changing the chemical composition of the atmosphere [24-27]. Depending on the region, biogeophysical and biogeochemical feedbacks of land cover on climate can amplify or dampen each other [28]. Through the land-atmosphere interactions, changes of the land cover and land use due to natural influence and policy induced land management alter weather and climate, hence can lead to the enhancement or reduction of the projected climate change signals expected from increased atmospheric CO_2_ concentration [25,29,30]. Past land use decisions have been shown to influence the mitigation potential in the boreal regions [31]. Depending on the carbon sequestration of the land cover, the CO_2_ warming of deforestation can dominate over albedo cooling effect (forests masks snow, which result in lower albedo). Several studies have addressed the biogeophysical cooling and moistening effect of tropical forests [29,32]. Whereas the magnitude of the net climate forcing and benefit of temperate forests and their role in the climate change mitigation is considered marginal or uncertain [32-34]. Climate model studies for the temperate region often show contradictory results. Replacing temperate forests with agriculture or grasslands can lead to lower surface air temperatures in summer [35,36] and may reduce the number of hot days [37]. In Canadian and Hungarian areas at the forest-steppe border forests showed a cooling and moistening effect on climate, thus may contribute to the drought mitigation [38,39]. These results indicate that climatic effects of forests are determined by various contrasting feedbacks. The variability of the climatic, soil and vegetation characteristics of a region, the length of analysed time scale [40], as well as the representation of land surface processes in the applied climate model, also have an influence on the simulated vegetation–atmosphere interactions.

Europe is the only continent with a significant increase of forest cover in recent times. In the last two decades the annual area of natural forestation and forest planting amounted to an average of 0.78 million hectares/year [41]. Land use and land cover change could be a very important driver for future environmental changes. The climatic feedbacks of land cover changes in Europe due to climate change and regional land use policies as well as the role of forests in the climate change mitigation are still poorly understood. The EC-FP7 project CC-TAME (Climate Change – Terrestrial Adaptation and Mitigation in Europe) aimed to prepare fine-scale studies not only for the assessment of the climate protecting effects of forests, but also for the development of adaptation strategies in forestry, agriculture and water management for the next decades. In order to contribute to this scientific goal, we prepared a case study to assess

• the biogeophysical effects of a hypothetic potential afforestation on summertime temperatures and precipitations, for the end of the 21st century and its regional differences within Europe,

• the magnitude of the biogeophysical feedbacks of forest cover increase compared to the projected climate change signal with special focus on the probability and severity of temperature and precipitation extremes.

## Results and discussion

### Methods overview

This subsection summarizes the most important aspects that are essential for the appropriate interpretation of the results. The experimental set-up and the method of the analyses are introduced in Sect. 4 more in detail.

**Figure 1:**
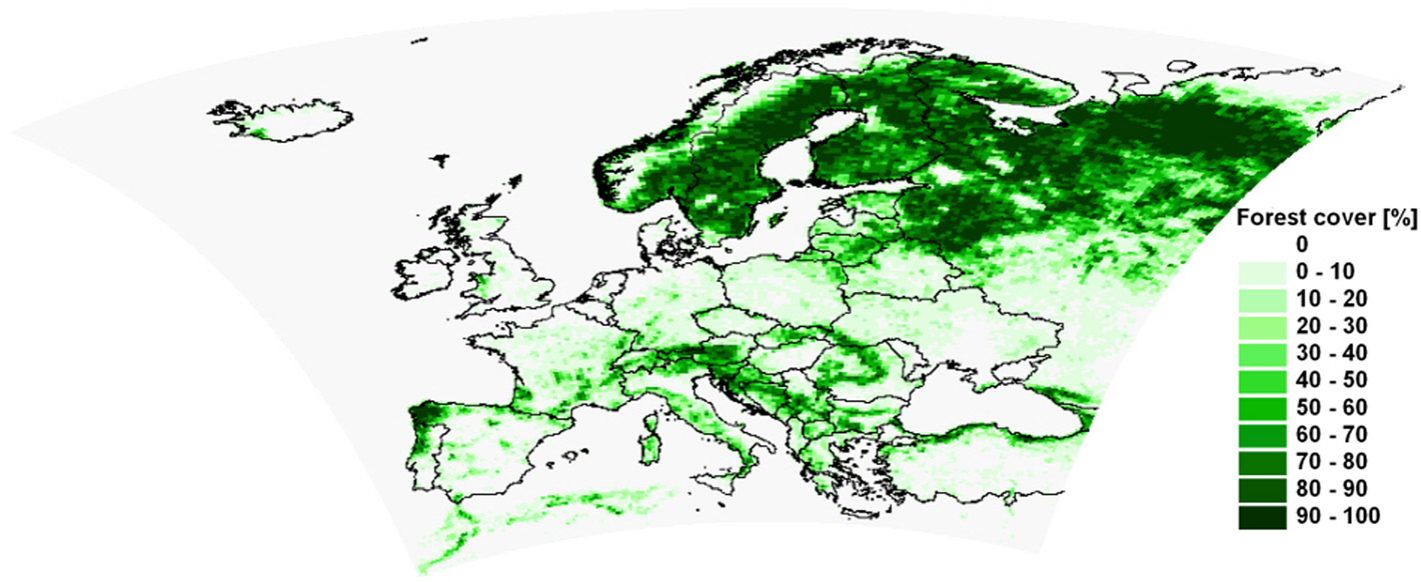
**Simulation domain with the present forest area in the model.** Horizontal resolution: 0.22°.

**Figure 2:**
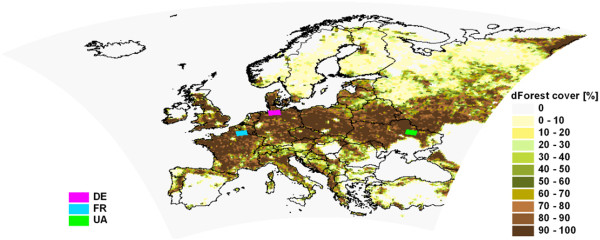
**Increase of the forest cover in the potential afforestation simulation compared to the present forested area in the model.** The three analysed regions are marked: Northern Germany (DE), Northeast France (FR) and Northeast Ukraine (UA).

In order to provide climate change information due to emission change, an emission scenario simulation for the future (2071–2090) and a reference simulation for the past (1971–1990) has been carried out applying the regional climate model REMO [42,43]. Both of them were performed with present (unchanged) forest cover (Table 1, Figure 1). To quantify the sensitivity of the model to changes in land cover, a hypothetic potential afforestation simulation has been prepared for the period 2071–2090 (Table 1, Figure 2). The analyses of the simulation results focused on the biogeophysical effects of forest cover increase on precipitation and temperature means and extremes in the summer months (June, July, August).

**Table 1:** Analysed data and time periods

**Experiment**	**Reference simulation**	**Potential afforestation simulation**
**Characteristics**	Present forest cover unchanged	Deciduous forests cover all additional vegetated area
**Time period**	1971–1990	2071–2090
	2071–2090	
**Greenhouse gas forcing**	IPCC-SRES emission scenario A2	
**Horizontal resolution**	0.22°	
**Lateral boundaries**	ECHAM5/MPI-OM^a^	

^a^ Roeckner et al. 2006, Jungclaus et al. 2006.

### Biogeophysical effects of emission change and potential afforestation on the summer temperature means and precipitation sums in Europe

**Figure 3:**
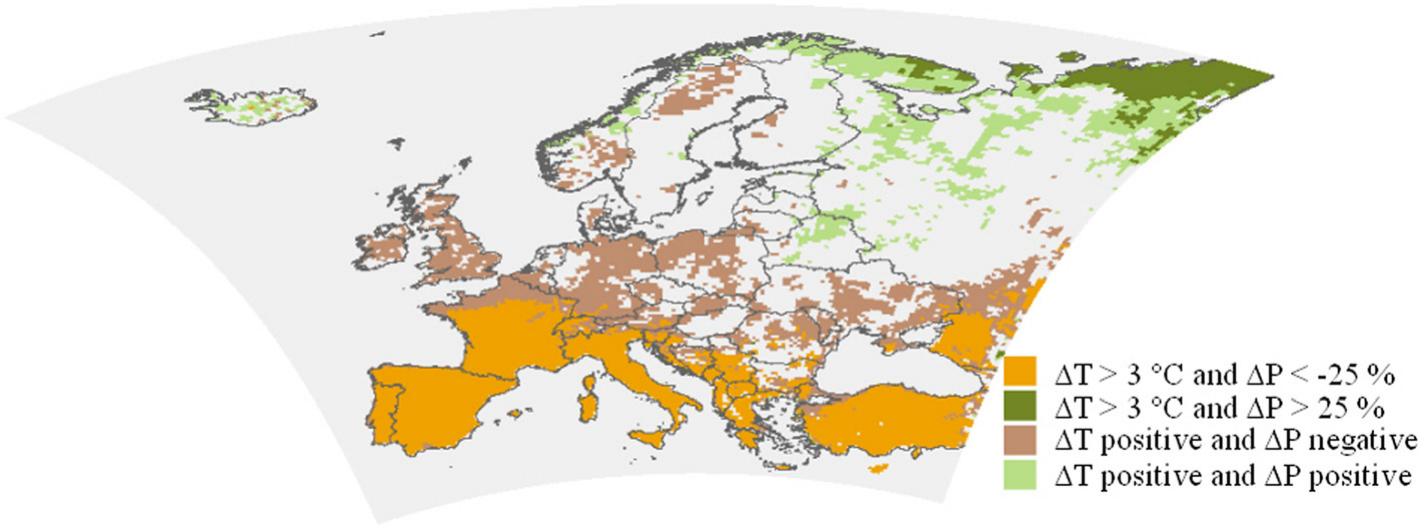
**The mostly climate change affected regions due to emission changes (∆ T: temperature change, ∆ P: precipitation change 2071–2090 vs. 1971–1990).** Only those regions are coloured, which are significant for ∆ T and ∆ P at 90% confidence level.

First, the sign and magnitude of the climate change signals without any land cover change have been investigated comparing the summer temperature means and precipitation sums in the time period 2071–2090 to 1971–1990. Increase of temperature is projected to occur with precipitation decrease in Southern- and Central-Europe and in the southern part of Scandinavia, whereas Northeast-Europe can be characterized with warmer and wetter conditions (Figure 3). In agreement with the results of other regional climate model simulations for Europe, the strongest warming and drying are expected in the Mediterranean area, southern France and over the Iberian Peninsula (Figure 3).

**Figure 4:**
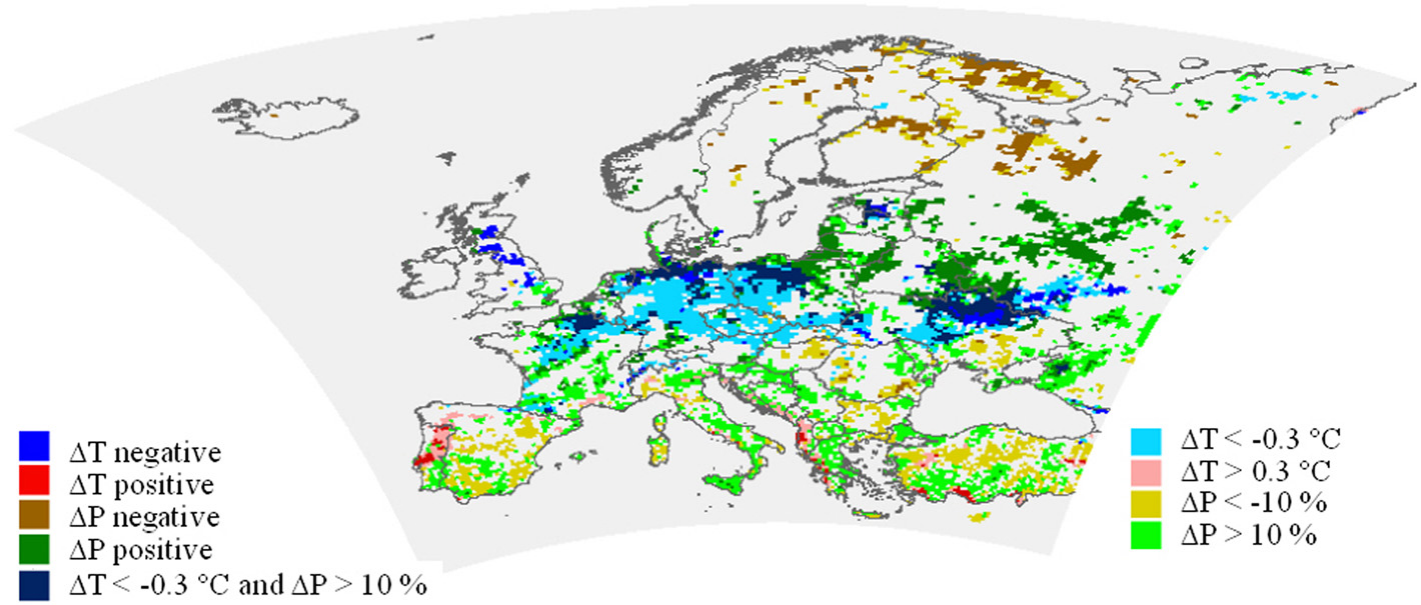
**The regions characterized by the largest effects of forest cover increase on temperature and precipitation (∆ T: temperature change, ∆ P: precipitation change for 2071–2090, without any change in emission).** The dark colour bar on the left side refers to significant changes, whereas the light colour bar on the right side to large but non-significant changes.

Second, climate change signal due to potential afforestation has been determined comparing the simulation results with- and without forest cover increase for 2071–2090. The regions have been identified, where the hypothetic forest cover increase shows the largest effects on summer temperature and precipitation (Figure 4). Land cover change affects the near-surface energy fluxes. The larger leaf area index and low aerodynamic resistance of forests (through increased roughness length) compared to other vegetated surfaces support the more intense vertical mixing. It leads to enhanced ability of evapotranspiration, thus to larger latent heat flux (not shown) and cooler surface temperatures. In northern part of Central-Europe and in Ukraine temperatures decreased by 0.3–0.5°C additionally to more than 10% increase (approx. 50 mm) of the summer precipitation sum in the potential afforestation simulation compared to the reference experiment with unchanged land cover (Figure 4). The precipitation conditions are also influenced by large-scale atmospheric circulation patterns, thus the precipitation signal is not linearly correlated with the amount of forest cover increase. In some boreal areas a relative small rate of afforestation resulted in a significant decrease of precipitation.

Consequently, the regions characterized by largest climatic effects of afforestation do not correspond to the areas with the largest signals due to emission change. The magnitude of the climatic effects of both emission change and potential afforestation differs among regions. In most parts of the temperate zone the cooling and moistening effects of afforestation are dominant during summer. These feedbacks can reduce the projected warming and drying especially in the northern part of Central-Europe and Ukraine. Whereas increase of the forest cover can enhance the climate change signal for precipitation in some part of Spain, Belarus and Russia but the magnitude of this impact is relatively small compared to the effect of the emission changes. Thus the analysis of the magnitude of the climatic feedbacks of afforestation relative to the effects of the enhanced greenhouse gas emission can help to determine the regions, where forests can play an important role in altering the climate change signal.

**Figure 5:**
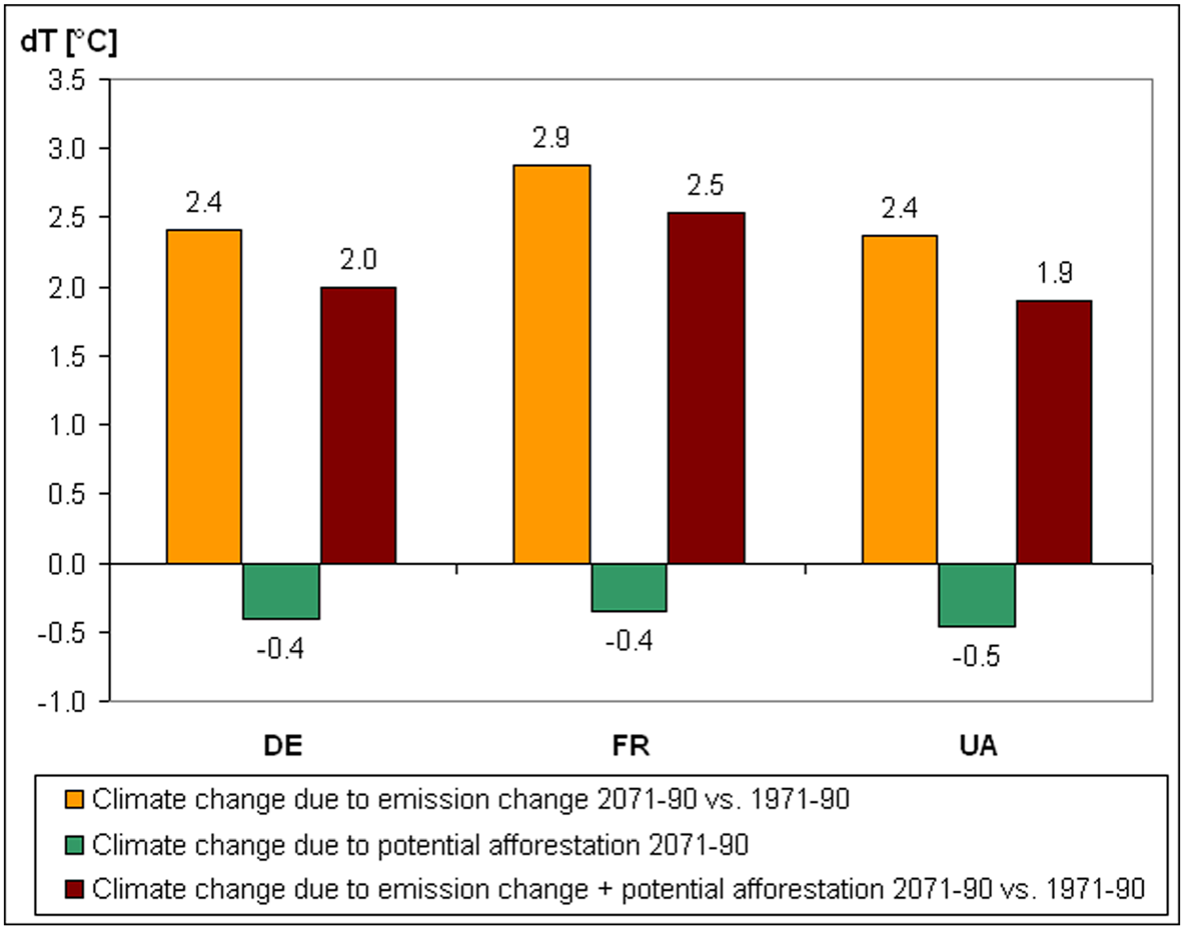
**Change of the summer temperature mean (∆ T) due to emission change (2071–2090 vs. 1971–1990), due to potential afforestation (2071–2090) and due to emission change + potential afforestation.** DE: Northern Germany, FR: Northeast France, UA: Northeast Ukraine.

**Figure 6:**
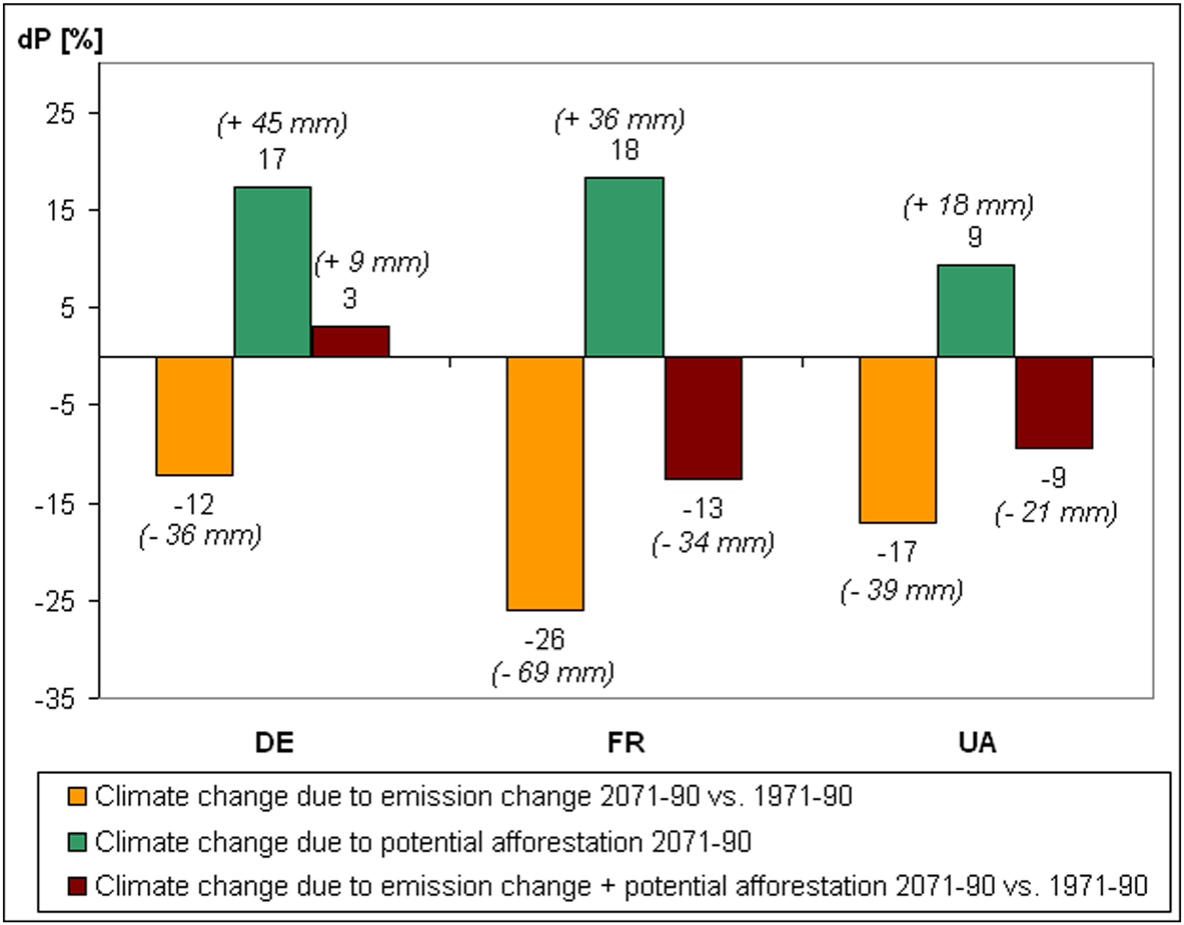
**Change of the summer precipitation sum (∆ P) due to emission change (2071–2090 vs. 1971–1990), due to potential afforestation (2071–2090) and due to emission change + potential afforestation.** DE: Northern Germany, FR: Northeast France, UA: Northeast Ukraine.

The regional characteristics of the effect of the assumed potential afforestation on temperature and precipitation have been analysed for three selected regions (Northern Germany: DE, Northeast France: FR, Northeast Ukraine, UA; Figure 2). Figures 5– 6 show that for both temperature and precipitation the climate change signal due to emission change and due to potential afforestation have the opposite sign. It means that climatic effects of emission change can be reduced by the forest cover increase in the selected regions. The temperature change signals for potential afforestation (−0.4 - -0.5°C) are smaller than for emission changes (+2.4 - +2.9°C). In northeastern part of Ukraine 20% of the emission change signal could be mitigated by the assumed afforestation (Figure 5).

The magnitude of the precipitation change shows larger spatial differences. In the northern part of Germany, the increase of the summer precipitation sum due to potential afforestation (+ 17%; + 45 mm) would be larger than its decrease due to the enhanced greenhouse gas emission (Figure 6). Thus the increase of forest cover would fully compensate the projected climate change signal, as long as there is enough soil moisture available. The combined effect of afforestation and emission changes for 2071–2090 would result in a net precipitation increase compared to the reference simulation for the past (1971–1990) without any land cover change.

In the region of Northern France, the precipitation decrease based on the A2 emission scenario is projected to be larger (−26%; -69 mm). If emission changes occurred together with potential afforestation, the half of the original climate change signal could be relieved (Figure 6). The relative climate change mitigating effect of potential afforestation is projected to be similar in Northern Ukraine (Figure 6), however both climate change signal and afforestation effect are smaller in this area.

### Effects of emission change and potential afforestation on the summer temperature and precipitation extremes

**Figure 7:**
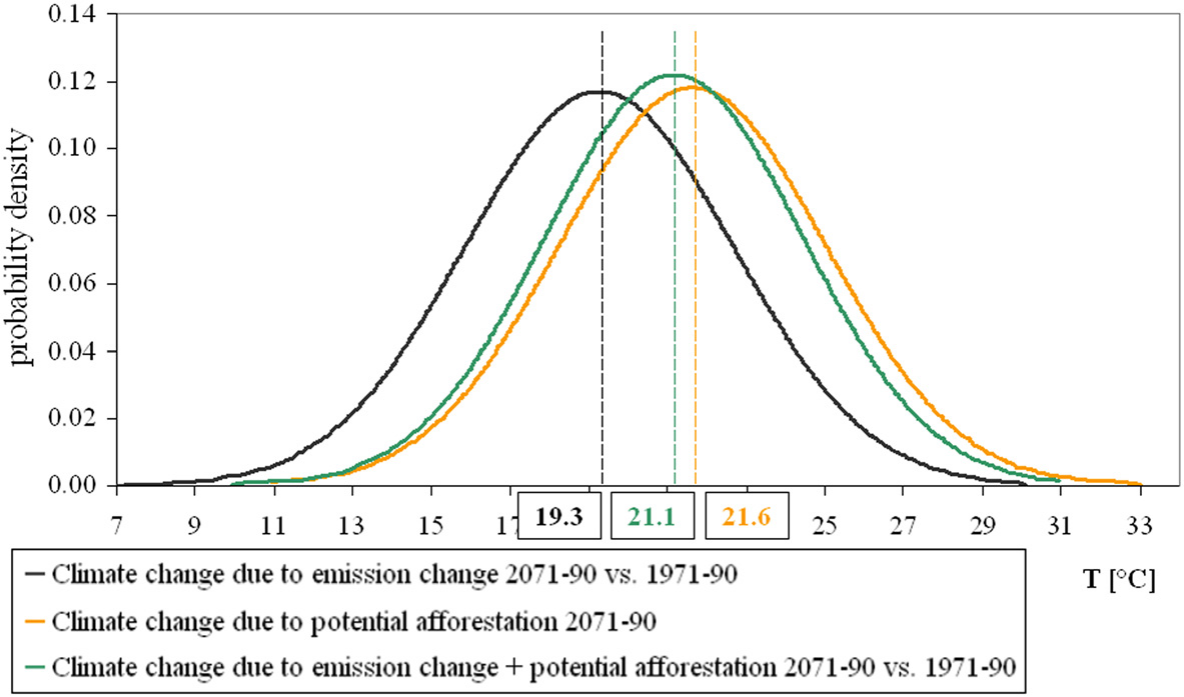
Probability density function of the daily temperature means (T) in the Northeast Ukrainian region.

Increase of forest cover affects not only the climatic means but also the extremes. The probability density functions (PDFs) of temperature show that distributions of the daily temperature means are shifted towards the warmer direction under future climate conditions (Figure 7). The PDF for the Ukrainian region indicates that the probability and severity of extreme warm summers may increase significantly under enhanced climate change. The PDF of the potential afforestation scenario shows a similar shape but with a slight shift towards colder values and a reduced upper tail compared to the reference in 2071–90 (the other two regions show similar behaviour – not shown). Consequently, increase of forest cover can result in cooler summer mean temperature (−0.5°C in Northern Ukraine) and may contribute to the decrease of temperature variability, thereby to the reduction of the projected climate change signal.

In each of the selected regions the total number of warm extremes (summer days, hot days, extremely hot days) are projected to increase significantly at the end of the 21st century (Table 2). Changes due to potential afforestation have the opposite sign but they are relatively small compared to the effect of the emission changes. The largest benefit of forest cover could be reached in the French region. Here, almost half of the increase in the number of extremely hot days could be mitigated by the assumed potential afforestation (Table 2).

**Table 2:** **Total number of the daily temperature and precipitation extremes **[ [44]]** for summer in the investigated 20-year time periods**

**Extreme index**	**Definition [unit]**	**Region**	**Number of days**	**Change of the number of days**		
			**REF**	**SA2 vs. REF**	**SA2F vs. SA2**	**SA2F vs. REF**
**SU**	when	**DE**	168	+212	−29	+183
Number of summer days	Tmax ≥ 25°C [day]	**FR**	248	+365	−32	+333
		**UA**	760	+382	−55	+327
**Tx30GE**	when	**DE**	21	+54	−20	+34
Number of hot days	Tmax ≥ 30°C [day]	**FR**	24	+152	−26	+126
		**UA**	151	+227	−31	+196
**Tx35GE**	when	**DE**	0	+2	−1	+1
Number of extremely hot days	Tmax ≥ 35°C [day]	**FR**	1	+22	−10	+12
		**UA**	8	+47	−7	+40
**RR1**	when	**DE**	796	+124	−53	+71
Number of dry days	Rday < 1 mm [day]	**FR**	931	+185	−45	+140
		**UA**	1098	**+132**	**−68**	**+64**
**RR10**	when	**DE**	133	**−13**	**+41**	**+28**
Number of heavy precipitation days	Rday ≥ 10 mm [day]	**FR**	127	**−38**	**+20**	**−18**
		**UA**	110	−21	+14	−7
**RR20**	when	**DE**	19	**0**	**+17**	**+17**
Number of very heavy precipitation days	Rday ≥ 20 mm [day]	**FR**	16	**+1**	**+10**	**+11**
		**UA**	14	**+3**	**+6**	**+9**

REF: Reference simulation 1971–90, SA2: Emission scenario simulation 2071–90, SA2F: Potential afforestation experiment 2071–90. DE: northern Germany, FR: northeast France, UA: northeast Ukraine. **Bold and scored values:** potential afforestation would reduce more than half of the climate change signal. **Bold values:** potential afforestation would enhance the climate change signal.

**Figure 8:**
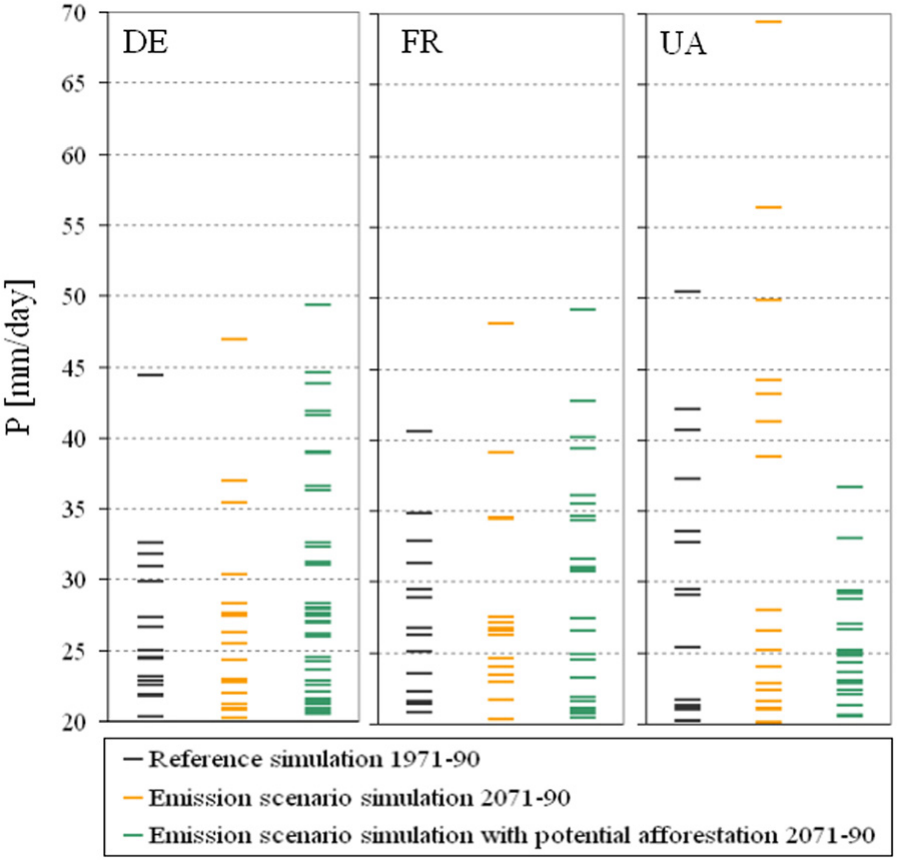
**Daily precipitation sums (P) in the summer months within the investigated 20-year time periods.** DE: Northern Germany, FR: Northeast France, UA: Northeast Ukraine.

Figure 8 illustrates that despite of the decrease of the summer precipitation sum, the probability of the extremely large daily precipitation amounts may increase by the end of the 21st century, especially in Ukraine. Assuming potential afforestation in this region, this tendency could be mitigated. The distribution of the precipitation amounts above 20 mm/day as well as the effects of emission and land cover change show spatial differences among the selected regions. In Northern Germany and Northern France, afforestation may enhance the effects of increased greenhouse gas concentrations, resulting in more severe precipitation events.

Analysing the selected WMO-CCL/CLIVAR extreme indices [44] for all summer days in the 20-year time periods (Table 2) it can be concluded, that under enhanced greenhouse gas conditions the number of dry days may increase. Potential afforestation would result in an increase of the daily precipitation amount. Thus the probability of dry days would decrease as well as the number of days characterised by larger than 10 mm precipitation may increase (Table 2). The latter could fully compensate the effect of emission change in the German region. In this area the total number of very heavy precipitation days show no changes due to emission change, but would increase by 17 due to potential afforestation (Table 2). In Northern Germany and Northern France not only the probability but also the severity of heavy precipitation events would increase assuming potential afforestation (Figure 8).

## Summary and conclusions

A case study has been prepared with the regional climate model REMO to assess the biogeophysical effects of a hypothetic potential afforestation scenario during summer in Europe for the end of the 21st century. Results of the A2 IPCC-SRES emission scenario simulations with and without forest cover increase have been compared to each other, in order to quantify the sensitivity of the regional climate model to land cover changes. For precipitation and temperature means and extremes, the sign and the magnitude of the biogeophysical effects of afforestation have been analysed relative to the climate change signal due to emission change. The regional characteristics of the effects have been investigated in three selected areas.

Results of the sensitivity study can be summarised as follows:

• In the temperate region potential afforestation can result in a decrease of the summer temperature mean (0.3-0.5°C) and an increase of the summer precipitation sum (up to 50–60 mm).

• For precipitation, the climate change mitigating effects of afforestation differs among the selected regions. In the northern part of Germany the increase of forest cover would fully compensate the projected climate change signal. In Northern France the precipitation decrease based on the A2 emission scenario is projected to be larger than in Northern Ukraine. In both regions the half of the climate change signal could still be relieved assuming potential afforestation.

• In each of the selected regions increase of forest cover may contribute to the decrease of the variability of the daily temperature means, thereby to the reduction of the projected climate change signal. The strong increase of the number of warm extremes (summer days, hot days, extremely hot days) due to emission change can be slightly reduced by the assumed potential afforestation.

• In Northern Germany and France, the forest cover increase would enhance the effects of emission change on extreme precipitation, resulting in more severe heavy precipitation events. The probability of dry days would decrease.

The magnitude of the possible climate change reducing effects of a potential afforestation for Europe, on regional scale, for longer future time period have not assessed before. Based on the simulation results it can be concluded that large, contiguous forest blocks can have distinctive biogeophysical effect on the climate on regional and local scale. Our land cover change study confirm that in smaller areas the biogeophysical feedback processes can significantly affect and modify the weather and climate, the temperature and precipitation variability [45,46]. The magnitude of the climatic effects of afforestation shows large spatial differences. Although even the hypothetic, practically unrealistic increases of forest cover could not offset the projected climate change in the most affected South-European regions, ecological services and local scale benefits of forest cover are highly valued. In the northern part of the temperate zone forests may play an important role in reducing the expected warming and drying during summer. Northern Germany is a relative humid region. Here, afforestation shows large climatic effects, as long as there is enough soil moisture available. The limiting role of the available soil moisture during the summer months has recently been investigated for this area for shorter time period (Petersen pers. comm.).

For the introduced sensitivity study, one regional climate model has been applied driven by one emission scenario. Multimodel ensembles and intercomparison studies are needed for studying the robustness of the results, which is the aim of recent EU-projects (e.g. LUCID; [47]). The spatial and temporal changes of vegetation cover due to climate change were not considered. So far, there is no information available about the climate change effects on the distribution of forest in Europe beyond limited case studies.

Our sensitivity study focused on the biogeophysical feedbacks, the biogeochemical interactions, the processes related to the carbon sequestration of forests and soil were not taken into account. In the temperate zone, net climatic effects of forests are determined by various contrasting feedbacks [29]. In case of biogeophysical processes, trees may contribute to warming due to their lower albedo relative to grass. But depending on regional characteristics forests can lead to cooling through the larger amount of evapotranspiration compared to other land surfaces. Similarly to Hogg et al. [38] Sánchez et al. [48], Wramneby et al. [30] and Gálos et al. [39], our simulations showed the dominant evaporative cooling effects for the entire summer period. However the results regarding the impacts of afforestation on temperature extremes are in contradiction with Anav et al. [37] for the same region. This result underlines that the simulated effects can largely depend on the description of the land surface properties and the representation of physical processes at the land surface and in the soil in the applied climate model [49]. Biogeophysical and biogeochemical effects can enhance or dampen each other. Forested areas sequester more carbon than grasslands. The carbon – climate feedbacks under future climate conditions are large unknowns [50]. Higher CO_2_ concentrations can also lead to the increase of the stomatal resistance thereby to the inhibition of the transpiration, which can amplify the global warming [51,52]. Therefore for the quantification of the net climatic benefits of forests, and to give appropriate suggestions for carbon management options an integrated assessment of these processes would be essential.

From a practical point of view, results of this case study related to the investigation of the climate sensitivity due to a hypothetic land use change and its regional differences can contribute to the future adaptation strategies in European agriculture and forestry. The understanding of the role of land cover in the climate system becomes even more important. Land cover characteristics due to climatic conditions as well as policy induced land management are region-specific. The sign and magnitude of the climatic effects of afforestation and emission change also shows large spatial differences. Therefore, to obtain regional scale information, similar fine scale case studies are essential to quantify and predict the climatic effectiveness of the different land cover and land use practices.

## Model and methods

### The regional climate model REMO – general characteristics and land surface representation

REMO (regional climate model at the Max Planck Institute for Meteorology; [42,43]) is a regional three-dimensional numerical model of the atmosphere. The calculation of the prognostic variables is based on the hydrostatic approximation. The physical parameterizations are based on the global climate model ECHAM4 [53]. Land surface processes in REMO are controlled by physical vegetation properties. The parameters of leaf area index and fractional vegetation cover for the growing and dormancy season, background albedo, surface roughness length due to vegetation, forest ratio, plant-available soil water holding capacity and volumetric wilting point are allocated to the different land cover types of the Olson distribution [54,55]. The parameters are aggregated to the model grid cell in the given horizontal resolution. The vegetation parameters can be linearly averaged, weighted by the fractional areas of the component land cover classes [56]. The only exception is the roughness length due to vegetation, which has to be logarithmically averaged at a so-called blending height [57]. In the current model version the vegetation phenology is represented by monthly varying values of leaf area index and vegetation ratio [58]. The mean climatology of the annual cycle of background albedo is also implemented [59,60]. All other land surface parameters remain constant throughout the year. REMO has been validated for Europe [43] and the simulation results have been compared to an ensemble of regional climate model projections [61].

### Experimental set up

The simulations have been carried out for Europe (Figure 1), with 0.22° horizontal grid resolution. REMO was driven with lateral boundary conditions from a simulation conducted with the coupled atmosphere–ocean model ECHAM5/MPI-OM [62,63].

The following experiments have been performed and analysed (Table 1):

• *Reference simulation* for the past (1971–1990) with present (unchanged) forest cover.

• *Emission scenario simulation* for the future (2071–2090) with unchanged forest cover applying the A2 IPCC-SRES emission scenario (continuously increasing global population and regionally oriented economic growth that is more fragmented and slower than in other storylines [64]). This experiment was the reference simulation to the land cover change study.

• *Emission scenario simulation with potential afforestation* for 2071–2090. The potential afforestation map (Figure 2) is based on the net primary production map for Europe derived from remotely sensed MODIS (Moderate-Resolution Imaging Spectroradiometer) products, precipitation and temperature conditions from the Wordclim database and soil conditions from the International Institute for Applied Systems Analysis.

Based on these conditions, areas on Figure 2 could be theoretically forests. However, land cover is also influenced by the land use policy, therefore the afforestation scenario in our study is a hypothetic one, where additional forested areas were assumed to be deciduous.

**Figure 9:**
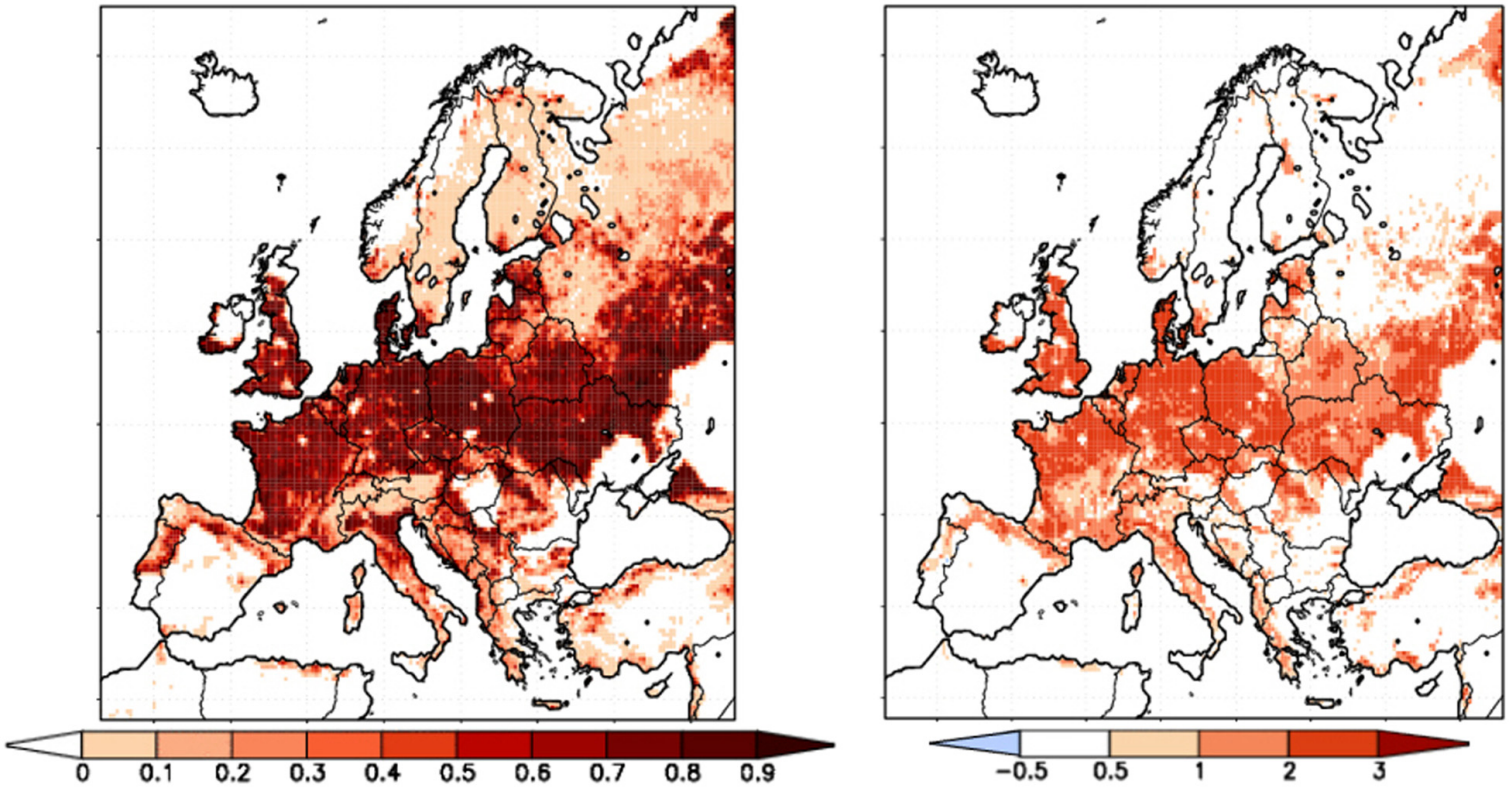
Changes of the roughness length ([m]; left) and leaf area index (right) for potential afforestation compared to the unchanged land cover (summer mean).

In case of the new potential forest cover map the fractional area of the forests has been increased. In order to include these changes into REMO, all characteristic land surface parameters (i.e. leaf area index and fractional vegetation cover for the growing and dormancy season, background albedo, surface roughness length due to vegetation, forest ratio, plant-available soil water holding capacity and volumetric wilting point) have been recalculated and reaggregated for all model grid cells. Figure 9 represents the changes of two selected land surface parameters, which play a determining role in the land-atmosphere interactions of the model. The increase of the forested area in Europe (Figure 2) corresponds to an increase of roughness length and leaf area index in summer (Figure 9).

### Method of analyses

The analyses of the simulation results focused on the summer months (June, July, August), because of the high radiation input, intense heat and mass exchange. The leaf area index of the deciduous forests reaches its maximum in this period, which has a strong control on the land-atmosphere interactions.

The sign and the magnitude of the temperature and precipitation changes have been analysed for the following three cases:

• Climate change due to emission change has been investigated comparing the results of the simulations with unchanged land cover for 2071–2090 to 1971–1990.

• Climate change due to potential afforestation have been calculated comparing the simulation results with- and without forest cover increase for the future time period (2071–2090).

• Climate change due to emission change and potential afforestation has been determined comparing the results of the potential afforestation experiment (2071–2090) to the reference study in the past (1971–1990) without land cover change.

A Mann–Whitney U-Test [65] was applied to test the significance of the climatic effects of afforestation and emission change. The regional characteristics of the effect of afforestation have been investigated for three selected regions in more detail, where temperature and/or precipitation changes are significant at the 90% confidence level and the assumed increase of the forested area exceeds 90%. The selected regions are (Figure 2): Northern Germany (DE), Northeast France (FR) and Northeast Ukraine (UA). All areas have the same size (15000 km^2^).

The probability distribution of temperature has been calculated from the daily mean values in the investigated 20-year time periods based on the normal distribution function. The indices of temperature and precipitation extremes in this study were selected from the list of climate change indices recommended by the World Meteorological Organization–Commission for Climatology (WMO–CCL) and the Research Programme on Climate Variability and Predictability (CLIVAR [44]). The selected indices (Table 2) describe cold and warm as well as wet and dry extremes. They are defined in terms of counts of days crossing absolute thresholds.

## Competing interests

The authors declare that they have no competing interests.

## Authors’ contributions

BG carried out the simulations, analyzed and interpreted the results and drafted the manuscript. GK provided the forest cover database and map for the potential afforestation case study. KS and CT provided expertise and guidance during the simulations. AH contributed to the statistical analysis and to the interpretation of the results. DR and SH participated in the design of the study and have been involved in the discussion of the results and the critical revision of the manuscript. DJ coordinated the research, participated in the design of the study and has given final approval of the version to be published. All authors read and approved the final manuscript.
